# Thermo- and Photoresponsive Smart Nanomaterial Based on Poly(diethyl vinyl phosphonate)-Capped Gold Nanoparticles

**DOI:** 10.3390/nano14191589

**Published:** 2024-10-01

**Authors:** Antonio Buonerba, Rosita Lapenta, Francesco Della Monica, Roberto Piacentini, Lucia Baldino, Maria Rosa Scognamiglio, Vito Speranza, Stefano Milione, Carmine Capacchione, Bernhard Rieger, Alfonso Grassi

**Affiliations:** 1 Department of Chemistry and Biology “*Adolfo Zambelli*”, University of Salerno, via Giovanni Paolo II, 84084 Fisciano, Italy; 2 CIRCC—Consorzio Interuniversitario per le Reattività Chimiche e la Catalisi, Villa La Rocca, via Celso Ulpiani 27, 70126 Bari, Italy; 3 Department of Biotechnology and Life Sciences, University of Insubria, via Jean Henry Dunant 3, 21100 Varese, Italy; 4 Department of Neuroscience, Università Cattolica del Sacro Cuore, 00168 Rome, Italy; 5 Fondazione Policlinico Universitario A. Gemelli IRCCS, 00168 Rome, Italy; 6 Department of Industrial Engineering, University of Salerno, via Giovanni Paolo II, 84084 Fisciano, Italy; 7 WACKER-Lehrstuhl für Makromolekulare Chemie, Zentralinstitut für Katalyseforschung (CRC), Technische Universitat München, Lichtenbergstraße 4, 85747 Garching, Germany

**Keywords:** thermoresponsive, photoresponsive, gold nanoparticles, LCST, FRET, smart, nanomaterial, poly(phosphonate), group transfer polymerization, yttrium

## Abstract

A new nanodevice based on gold nanoparticles (AuNPs) capped with poly(diethylvinylphosphonate) (PDEVP) has been synthesized, showing interesting photophysical and thermoresponsive properties. The synthesis involves a properly designed Yttriocene catalyst coordinating the vinyl-lutidine (VL) initiator active in diethyl vinyl phosphonate polymerization. The unsaturated PDEVP chain ending was thioacetylated, deacetylated, and reacted with tetrachloroauric acid and sodium borohydride to form PDEVP-VL-capped AuNPs. The NMR, UV–Vis, and ESI-MS characterization of the metal nanoparticles confirmed the formation of the synthetic intermediates and the expected colloidal systems. AuNPs of subnanometric size were determined by WAXD and UV–Vis analysis. UV–Vis and fluorescence analysis confirmed the effective anchoring of the thiolated PDEVP to AuNPs. The formation of 50–200 nm globular structures was assessed by SEM and AFM microscopy in solid state and confirmed by DLS in aqueous dispersion. Hydrodynamic radius studies showed colloidal contraction with temperature, demonstrating thermoresponsive behavior. These properties suggest potential biomedical applications for the photoablation of malignant cells or controlled drug delivery induced by light or heat for the novel PDEVP-capped AuNP systems.

## 1. Introduction

Thermoresponsive polymers are a class of smart materials characterized by a reversible phase transition in response to temperature variations. Polymers with a lower critical solution temperature (LCST) are soluble in water below the transition temperature and become insoluble above this temperature, leading to phase separation [1,2,3]. This property makes them highly valuable in biomedical applications where precise control over polymer behavior is critical [4]. Polymers showing LCST are characterized by the polymer chains’ contraction and the solvation molecules’ expulsion upon increasing temperature. This property can be exploited for thermal-triggered drug delivery [4]. Various polymerization techniques are employed to synthesize these polymers, including free radical polymerization, reversible addition–fragmentation chain transfer (RAFT) polymerization, atom transfer radical polymerization (ATRP), and ring-opening polymerization (ROP), allowing the creation of polymers with well-defined structures and narrow molecular weight distributions [5]. Common monomers used include *N*-isopropylacrylamide (NIPAM) to form PNIPAM with an LCST around 32 °C [6] and *N*-vinyl caprolactam (NVCL) leading to PNVCL with an LCST around 30–35 °C [7,8]. In biomedical applications, thermoresponsive polymers are extensively used in drug delivery systems that release their payload in response to temperature changes, such as PNIPAM-based hydrogels that release drugs when reaching a temperature above their LCST [4,6]. They are also valuable in tissue engineering, where they form scaffolds that respond to physiological temperature changes, aiding cell attachment and proliferation [9]. Additionally, these polymers are used in wound healing, where hydrogels made from thermoresponsive polymers conform to the wound site at body temperature and release therapeutic agents in a controlled manner [10,11]. Furthermore, thermoresponsive polymers are applied in diagnostics where their phase transition can trigger signal changes in biosensors [12]. Ongoing research in their synthesis continues to expand their potential, promising further innovations and improved healthcare outcomes.

Group transfer polymerization (GTP) is an important route for synthesizing thermoresponsive smart polymers [13,14]. GTP is a living polymerization technique that allows precise control over molecular weight and architecture, typically applied in acrylic and methacrylic ester polymerization. Lanthanide catalysts have garnered attention in GTP due to their high reactivity, ability to form stable complexes, and tolerance to various functional groups. These catalysts enable rapid polymerization, producing high-molecular weight polymers with narrow molecular weight distributions and well-defined structures. Lanthanide complexes of β-diketonate, phosphine oxides, or cyclopentadienyl ligands suit this purpose. The advanced materials synthesized by lanthanide-catalyzed GTP find applications in nanotechnology, electronics, materials science, and biomedical fields, particularly in drug delivery systems where uniform polymer size is crucial [14]. An advantage of GTP is the wide range of monomers that can be polymerized using this technique. Extensive research efforts enlarged the variety of monomers, which was initially limited to a,β-unsaturated carbonyl compounds; acrylates, acrylamides, vinyl phosphonates, and vinyl pyridines with numerous different functionalities have been successfully homo- and co-polymerized using various catalysts [14,15,16,17,18,19]. Rieger et al. mainly focused attention on the polymerization of phosphorous-containing polymers since they show high biocompatibility, water solubility, and potential for various biomedical applications [14]. The polymerization of diethyl vinyl phosphonate (DEVP) was, for the first time, obtained with Cp_2_YbCl (Cp = cyclopentadienyl) and Cp_2_YbCH_3_ catalysts with high activities yielding high-molecular weight polymers [14]. Later studies identified the Yasuda-type polymerization mechanism and introduced further catalytic systems; in particular, simple Cp_3_Ln (Ln = Lu, Yb, Tm) complexes led to well-controlled polymerization [20]. Rieger and co-workers reported the preparation of ex situ synthesized large gold nanoparticles (27.8 ± 7.5 nm) functionalized via click chemistry with poly(diethylvinylphosphonate) (PDEVP) and the UV–Vis and DLS characterizations [21].

**Figure 1:**
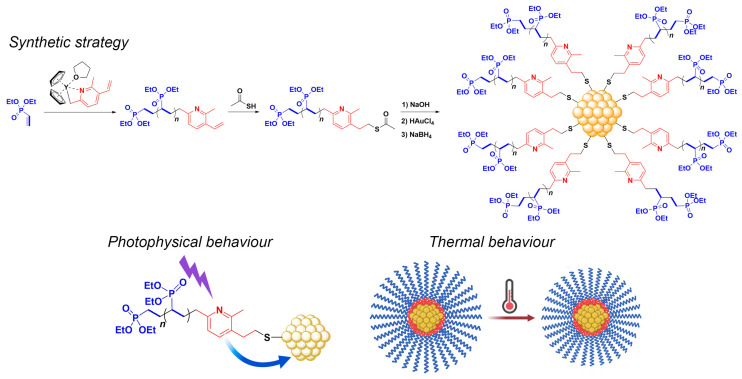
Synthetic strategy and photophysical and thermal behavior of P(DEVP)-capped AuNPs.

In this study, the synthesis of PDEVP initiated with vinyl lutidine (PDEVP-VL) was obtained with a novel Yttriocene catalytic system coordinating this initiator. The PDEVP-VL was later thioacetylated [22,23] (PDEVP-VL-TA) and subsequently deacetylated for anchoring to the in situ synthesized gold nanoparticles (PDEVP-VL-AuNPs) (Figure 1). The novel PDEVP-capped AuNPs were fully characterized and the thermal and photophysical behaviors were investigated. Here, we disclose that the synthetic strategy afforded ultra-small nanoparticles (≤2 nm) with novel photothermal properties. The complete characterization of the novel catalytic system, the (functionalized) polymers, and the resulting PDEVP-VL-S-capped AuNPs is reported. In addition, the thermal and photophysical behavior in aqueous media was disclosed for the first time. Finally, preliminary results on the photoablation of cells with pulsed near-infrared radiation (NIR) are presented to testify to these systems’ potential biomedical applications.

## 2. Materials and Methods

### 2.1. Materials and General Procedures

**Scheme 1:**
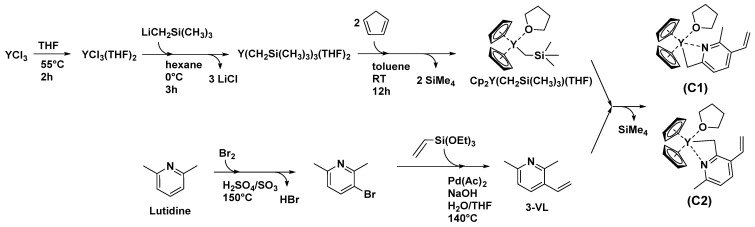
Synthetic procedure for the complexes Cp_2_Y(VL)(THF) (**C_1_**–**C_2_**).

Manipulations of air- and/or water-sensitive compounds were performed using Schlenk techniques with an MBraun glovebox under a protective nitrogen atmosphere. Commercial-grade toluene, hexane, and tetrahydrofuran (Sigma-Aldrich, Milan, Italy) were pre-dried over calcium chloride or potassium hydroxide, refluxed for 48 h under a nitrogen atmosphere over sodium, and distilled before use. The 2,6-lutidine (98%; Sigma-Aldrich), bromine (99.5%; Sigma-Aldrich), fuming sulfuric acid (Sigma-Aldrich), triethoxy(vinyl)silane (98%; TCI chemical, Zwijndrecht, Belgium), palladium acetate (98%; Sigma-Aldrich), yttrium (III) chloride anhydrous (99.9%; Sigma-Aldrich), (trimethylsilyl)methyllithium solution (1.0 M in pentane; Sigma-Aldrich), tetrachloroauric acid trihydrate (≥49.0% Au basis; Sigma-Aldrich), water (HPLC grade; Sigma-Aldrich), methanol (HPLC grade; Sigma-Aldrich), dichlomethane (HPLC grade; Sigma-Aldrich), pentane (99%; Sigma-Aldrich), thioacetic acid (96%; Sigma-Aldrich), benzophenone (99%; Sigma-Aldrich), sodium hydroxide (98%; Sigma-Aldrich), potassium hydroxide (85%; Sigma-Aldrich), sodium borohydride (95%, TCI chemical), and sodium chloride (99.8%; Sigma-Aldrich) were used as received without further purification procedures. Diethylvinylphosphonate (DEVP) was prepared according to the literature procedure [24], anhydrified with CaH_2_ and distilled before use. The 2,6-dimethyl-3-vinylpyridine was synthesized via bromuration of 2,6-dimethylpyridine to 3-bromo-2,6-dimethyl-pyridine according to Zimmermann’s procedure [25] and subsequent vinylation with triethoxy(vinyl)silane via Hiyama–Heck reaction promoted by palladium acetate, according to the procedure reported by Gordillo [26,27] (Scheme 1). The 2,6-dimethyl-3-vinylpyridine was purified by column chromatography using silica gel 60 as the stationary phase and hexane-ethyl acetate (*v*/*v* = 6:4) as the eluent (see Figure S1 for the NMR characterization). The Yttrium complexes di(cyclopentadienyl)((6-methyl-5-vinylpyridin-2-yl)methyl)yttrium(tetrahydrofuran) (**C_1_**) and di(cyclopentadienyl)((6-methyl-3-vinylpyridin-2-yl)methyl)yttrium(tetrahydrofuran) (**C_2_**) were synthesized according to the procedure depicted in Scheme 1. Y(CH_2_Si(CH_3_)_3_)_3_(THF)_2_ was synthesized according to Hultzsch’s procedure [28]. (C_5_H_5_)_2_Y(CH_2_Si(CH_3_)_3_)(THF) was synthesized according to the procedure reported by Salzinger [20]. Briefly, (C_5_H_5_)_2_Y(CH_2_Si(CH_3_)_3_)(THF) (1 eq.) in toluene was added to 2,6-dimethyl-3-vinylpyridine (1 eq.) at room temperature and the solvent was removed in vacuo.

#### 2.1.1. General Procedure for the Synthesis of Poly(diethyl vinyl phosphonate) (Referred to in Entry 1 of Table 1)

A round bottom flask (volume of 50 mL), at room temperature in a glove box under a protective nitrogen atmosphere, was equipped with a magnetic stir bar and charged with the Yttrium catalyst (mixture of the complexes C_1_–C_2_ as prepared, 0.305 mmol, 0.129 g), toluene (20 mL), and DEVP (12.1 mmol, 2.0 g, 40 eq.). After 1 h of stirring at room temperature, the polymer was coagulated in pentane and dried in a vacuum. The yield was 1.39 g.

#### 2.1.2. Thioacetylation of PDEVP

A round bottom flask (volume of 50 mL) equipped with a magnetic stir bar was charged with poly(diethyl vinyl phosphonate) (1.10 g, 0.043 mmol) dissolved in toluene (20 mL) at 80 °C. The solution was slowly cooled to room temperature, and thioacetic acid (0.033 g, 0.43 mmol, 10 eq. with respect to the polymer chain moles) and benzophenone (0.008 g, 0.043 mmol, 1 eq. with respect to the polymer chain moles) were sequentially added. The flask was transferred into a UV oven and irradiated at 365 nm for 4 h. The reaction mixture was then poured in plenty of pentane to coagulate thioacetylated poly(diethyl vinyl phosphonate), which was recovered by filtration and dried in a vacuum. The yield was 0.79 g.

#### 2.1.3. In Situ Synthesis of Thiolated-PDEVP and Formation of PDEVP-Coated Gold Nanoparticles

A round bottom flask (volume of 50 mL), equipped with a magnetic stir bar and a condenser, was charged with thioacetylated poly(diethyl vinyl phosphonate) (0.182 g, 0.0103 mmol) and 5 mL of methanol (5 mL). The solvent was refluxed until the dissolution of the polymer and then slowly cooled to room temperature. Sodium hydroxide (2.4 mg, 0.06 mmol) was added and left to react at room temperature for 30 min. Subsequently, a methanol solution of tetrachloroauric acid trihydrate was added (2.1 mg, 0.005 mmol, dissolved in 5 mL of methanol), followed by a methanol solution of sodium borohydride (0.025 mmol, 0.05 M). The reaction mixture became rapidly brownish in color and was left under stirring at room temperature overnight. The solvent was removed in a vacuum, and the product was dissolved in the minimum volume of dichloromethane and filtered with a PTFE syringe filter (pore size of 0.45 µm). The purified poly(diethyl vinyl phosphonate)-capped gold nanoparticles were dried in a vacuum, yielding 0.164 g.

#### 2.1.4. Cell Cultures

Co-cultures of murine neurons and astrocytes were created from E18 embryos of C57BL/6 mice as in Buonerba et al., 2020 [29]. Neuronal cells were maintained in a culture medium for 10 days before treatment with PDEVP-coated AuNP.

### 2.2. Instrumentation and Analytical Methods

Nuclear magnetic resonance (NMR) spectra were recorded with Bruker AVANCE spectrometers (600, 400, and 300 MHz for ^1^H NMR). Chemical shifts were referenced to tetramethylsilane as an external reference using residual protio signals of deuterated solvents. Ultraviolet–visible (UV–Vis) absorbance spectra were obtained with a Cary 50 spectrophotometer from Varian, Las Vegas, NV, USA. Fluorescence spectra were obtained with a Cary Eclipse fluorescence spectrophotometer from Varian. Electrospray ionization mass spectrometry (ESI-MS) spectra were acquired on a Quattro microTM API triple quadrupole mass spectrometer (MS) from Waters equipped with an electrospray ion (ESI) source. Fourier transform infrared (FT-IR) analyses were carried out on a Bruker Vertex 70 spectrometer equipped with a DTGS detector and a Ge/KBr beam splitter. The samples were analyzed while dissolved in dichloromethane solutions or embedded in potassium bromide disks. Wide-angle X-ray diffraction (WAXD) patterns were obtained in reflection mode with an automatic Bruker D8 powder diffractometer using the nickel-filtered CuKα radiation. Atomic force microscopy (AFM) micrographs of thin films were obtained in the air at room temperature with a Dimension 3100 module coupled with a Nanoscope V controller from Bruker operating in tapping mode. The films were obtained by depositing methanol polymer solutions (0.2 wt%) at room temperature onto glass slides. Commercial probe tips with nominal spring constants of 20–100 Nm^−1^, resonance frequencies in the range of 200–400 kHz, and tip radii of 5–10 nm were used. The AFM micrographs were analyzed with the NanoScope Analysis (v190R1sr2) software from Bruker, Ettlingen, Germany. Scanning electron microscopy (SEM) images were acquired employing a Zeiss Supra 50 field emission microscope from Carl Zeiss (Gottingen, Germany). The size of gold colloids was measured by dynamic light scattering (DLS) performed on a temperature-programmable Zetasizer Nano-ZS from Malvern Instruments, Malvern, UK. Thioacetylation reaction [22,23,30] was carried out in a UV incubator (Bio-Link BLX from Vilber Lourmat, Marne-la-Vallée, France) at a wavelength of 365 nm and power of 100 W.

### 2.3. Computational Details

DFT calculations were performed at the GGA level with the Gaussian 09 set of programs [31] using the BP86 functional of Becke and Perdew [32,33]. The electronic configuration of the molecular systems was described with the standard split valence basis set with a polarization function of Ahlrichs and co-workers for H, C, N, and O (SVP keyword in Gaussian) [34]. We used The LanL2DZ basis set for Y with associated effective core potentials [35]. Geometry optimizations were performed without symmetry constraints; the vibrational frequency calculations validated all the obtained structures as minima. The reported Gibbs energies have been obtained by adding thermal correction in the gas phase to the electronic energies in the solvent (SMD model) computed via single-point energy calculations in benzene at the BP86 level with the triple-z basis set of Ahlrichs (TZVP) for H, C, N, and O and the LanL2DZ ECP for Y.

### 2.4. Phototheral Effect

The photothermal effect was explored in neuronal cells that were previously treated for 24 h with PDEVP-coated AuNPs at a concentration of 700 µg/mL in the culture medium and irradiated by a pulsed laser set to a wavelength of 760 nm. For these measurements, we used a Mai Tai DeepSee™ eHP laser (Spectra Physics, Milpitas, CA, USA, 80 MHz, <70 fs pulse width, maximum average exit power 2.7 W). The laser operated at power levels ranging from 30 to 539 W/cm^2^. Power variations were achieved using a filter wheel positioned in front of the laser exit and connected to a Nikon A1MP confocal system (Tokyo, Japan) attached to a Nikon Eclipse Ti Series inverted microscope equipped with a 40× oil-immersion objective, limiting the transmittance. The single-frame duration was 1.26 s. Throughout the experiments, cells were maintained in Tyrode’s solution, and the emergence of heat-induced bubbles was observed in the aqueous culture medium.

## 3. Results and Discussion

### 3.1. Synthesis of the Yttriocene Catalysts C_1_ and C_2_

**Figure 2:**
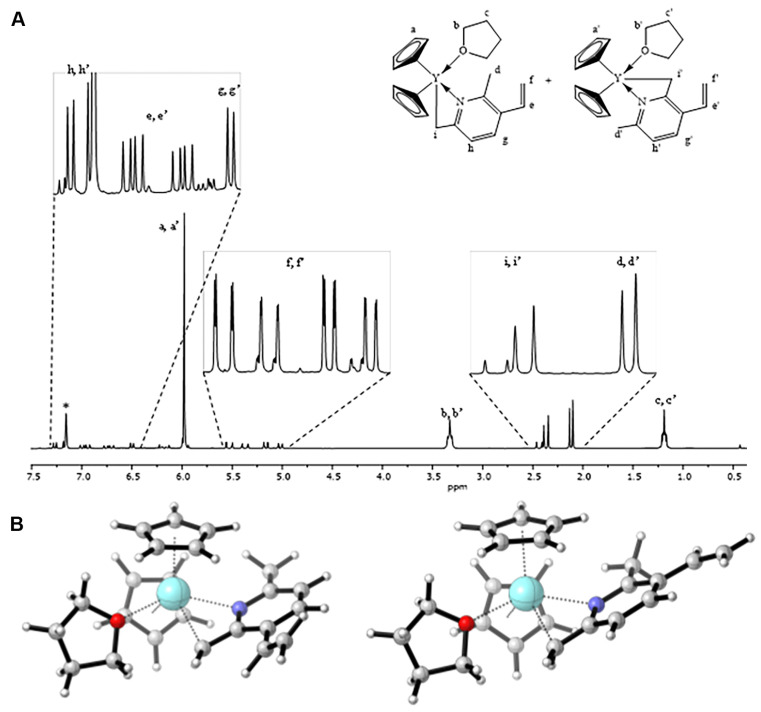
^1^H-NMR spectrum of Cp_2_Y(VL)(THF) (300 MHz, 25 °C, * = C_6_D_6_,) (**A**). Minimum energy structures for complexes C_1_ and C_2_ (**B**).

The pro-ligand 3-vinyl-lutidine (3-VL) was synthesized via bromuration of lutidine with bromine followed by Hiyama–Heck reaction with (vinyl)triethoxisilane catalyzed by Palladium diacetate (Scheme 1, Figure S1). The corresponding Yttriocene complexes of general formula Cp_2_Y(R)(THF) (R = (2-CH_3_)(3-C_2_H_3_)(6-CH_2_)C_5_H_2_N (**C_1_**); (2-CH_2_)(4-C_2_H_3_)(6-CH_3_) C_5_H_2_N (**C_2_**)) were obtained from the reaction of Cp_2_Y(CH_2_Si(CH_3_)_3_)(THF) with 3-VL using the modified literature procedure depicted in Scheme 1. The ^1^H NMR signals of THF are shifted upfield, suggesting THF coordination to the metal center and the monomeric structure of C_1_ and C_2_ in the THF solution. The ^1^H methylene protons at 2.0–2.5 ppm (Figure S2) bound to Yttrium in C_1_/C_2_ are split into two signals accounting for the presence of isomers of C_1_ and C_2_, probably dimeric species, in a slow exchange regime in the NMR time scale (Figure 2A). Accordingly, the same splitting was observed for the vinyl protons at ppm and the aromatic protons at 5.0–5.6 and 6.6–7.1 ppm of the 3-VL ligand.

The C_1_ and C_2_ complexes were obtained in the C_1_/C_2_ molar ratio of 1.1, thus showing a slightly higher reactivity of the methyl group in the 6-position of the 3-VL moiety in the course of the σ-bond metathesis reaction with Cp_2_Y(CH_2_Si(CH_3_)_3_)(THF). DFT calculations were undertaken to achieve a model for these complexes. The minimum energy structures of C_1_ and C_2_ are reported in Figure 2B. In both complexes, the Yttrium atom is set at the center of a distorted square pyramid, whose base is set by lutidine, THF, and one of the cyclopentadienyl rings. The lutidine is *k*^2^ bonded to the metal through the nitrogen and the carbon atoms. The bond distances are comparable to those reported for the similar Cp_2_Y(CH_2_(C_5_H_2_Me_2_N) dimeric complex. The two structures are very close in energy (DG = 0.5 kcal/mol).

### 3.2. Synthesis of PDEVPs End-Capped with Lutidine

**Figure 3:**
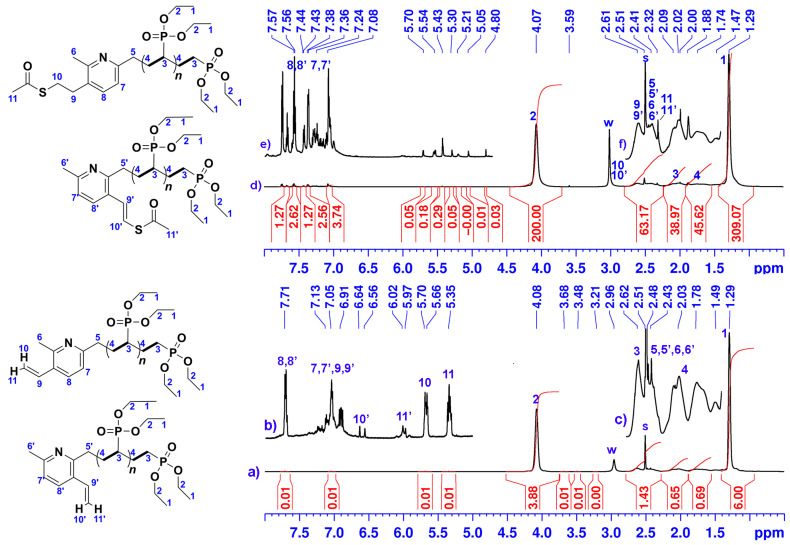
^1^H NMR spectra (600 MHz, DMSO-*d*_6_, 90 °C) of P(DEVP)-VL (**a**) and P(DEVP)-VL-TA (**d**) with the corresponding magnifications of the aromatic/vinyl (**b**,**e**) and aliphatic (**c**,**f**) spectral regions (w = water; s = residual protio signal of the deuterated solvent).

**Scheme 2:**
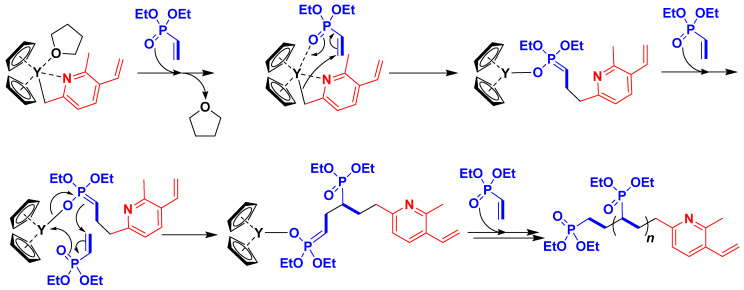
Group-transfer polymerization of DEVP catalyzed by **C1/C2**.

Group transfer polymerization (GTP) of diethyl-vinyl-phosphonate (DEVP) catalyzed by the C_1_/C_2_ mixture yielded poly(diethyl-vinylphosphonate)s (PDEVPs) with a narrow polydispersity index containing substituted lutidine chain endings (Scheme 2). The polymerization runs were performed at different monomer-to-catalyst ratios to produce PDEVPs with different molecular mass. The *M*_n_ value of the synthesized PDEVPs was determined by integration of the ^1^H NMR signals (Figure 3a) of the aromatic protons in the 3-VL chain ending with the ^1^H NMR methyl signal of the monomer in the main chain; the corresponding data are given in Table 1. Figure 3a and Figures S2–S4 report the complete NMR assignments for the VL-terminated PDVPs (PDEVP-VL). Polymerization reactions at the very low monomer-to-catalyst ratio were carried out to assess the initiation efficiency and selectivity of the first insertion reaction of DEVP (Table 1). The ESI-MS spectrum of the oligomers shows that the initiation reaction is fast and selective where the monomer insertion occurs on the 3-VL initiator (Figure S6). The spacing between the mass peaks is 165.1 g/mol, namely the DEVP molecular weight, and the absolute mass is due to the addition of *n* units of DEVP to the 3-VL initiator. The amplitude of the mass peak distribution is like that of PVDEP oligomers resulting from CGP of DEVP catalyzed by Yttriocenes. The ^1^H-NMR spectrum of the PVDEPs after coagulation and washing with solvents confirms this attribution; one prevalent pattern of ^1^H signals for the vinyl group and aromatic protons was observed, suggesting that the insertion of the first DEVP units selectively occurs on one of the two stereoisomers, likely C_1_ as a result of the similar chemical shifts of the ^1^H NMR vinyl signals in the polymer and the C_1_ catalyst.

### 3.3. Synthesis of PVDEP-VL-Capped AuNPs

**Scheme 3:**
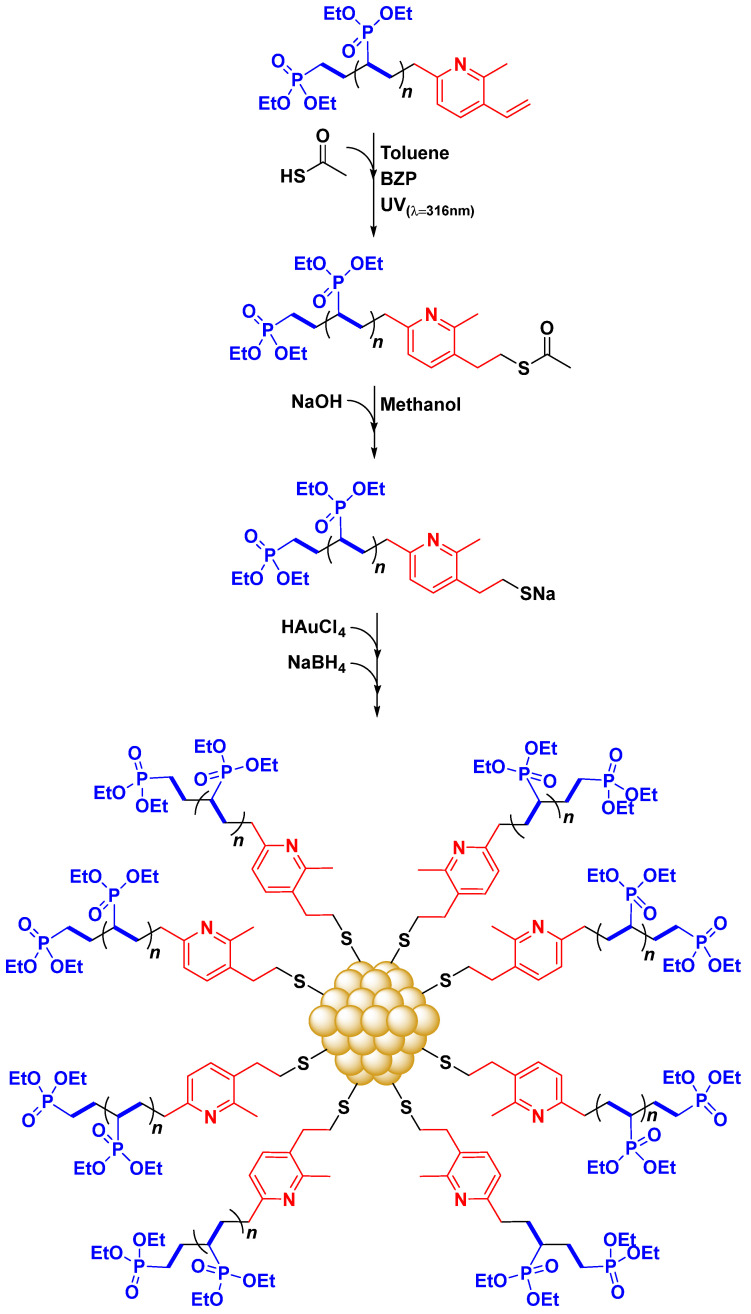
Synthetic strategy for the P(DEVP) anchoring to AuNPs.

PDEVP-VLs were converted into the corresponding thioacetylated derivatives (PDEVP-VL-TA) by radical addition of thiolacetic acid to the vinyl group of 3-VL under UV irradiation in the presence of benzophenone (Scheme 3) [22,23,30]. The resulting polymer was characterized by NMR spectroscopy (Figure 3b and Figure S5) and FT-IR spectroscopy (Figure S7). PDEVP-VL-TA was dissolved in methanol and treated with an excess of sodium hydroxide to produce a polymer ending with thiolate functionality (PDEVP-VL-S-Na). Finally, a methanol solution of HAuCl_4_ was treated at room temperature with an excess of NaBH_4_ in the presence of PDEVP-VL-S-Na and left under stirring overnight. Gold nanoparticles coated with PVDEP corona (PVDEP-VL-S-AuNPs) were recovered by filtration and dried in a high vacuum, producing a deep-colored solid.

**Figure 4:**
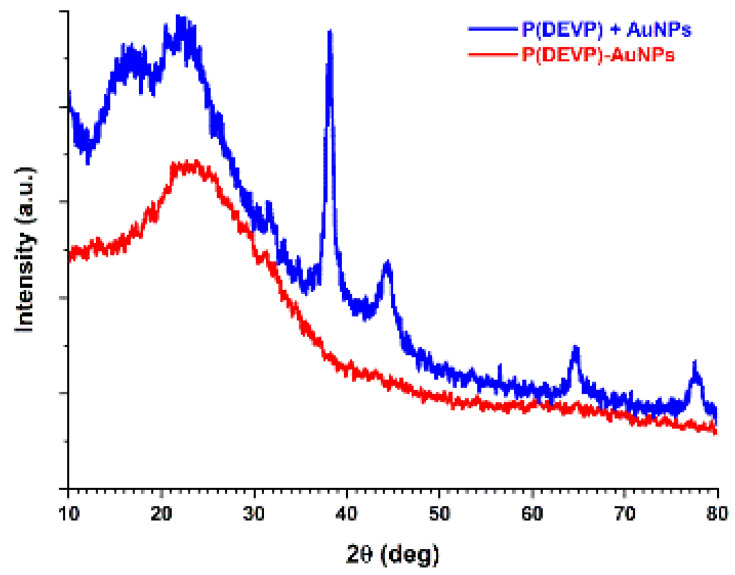
WAXD patterns of P(DEVP)-capped AuNPs (red curve) and AuNPs synthesized in the presence of P(DEVP) (blue curve).

**Figure 5:**
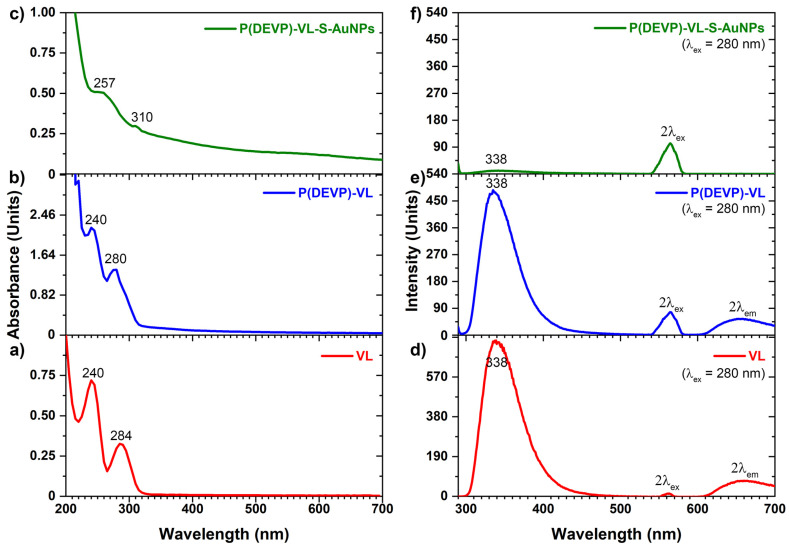
UV–Vis (panels (**a**–**c**)) and fluorescence emission spectra (panels (**d**–**f**); excitation at a wavelength of 280 nm) of vinyl-lutidine (1.6 × 10^−2^ mM; panels (**a**,**d**)), P(DEVP)-VL (0.45 mg/mL; panels (**b**,**e**)), and P(DEVP)-VL-S-AuNPs (0.45 mg/mL; panels (**c**,**f**)). Bands centered at 560 nm (2λ_ex_) and 676 nm (2λ_em_) in fluorescence emission spectra (**d**–**f**) are due to 2nd order diffraction phenomena by the grating of the spectrometer monochromator.

**Figure 6:**
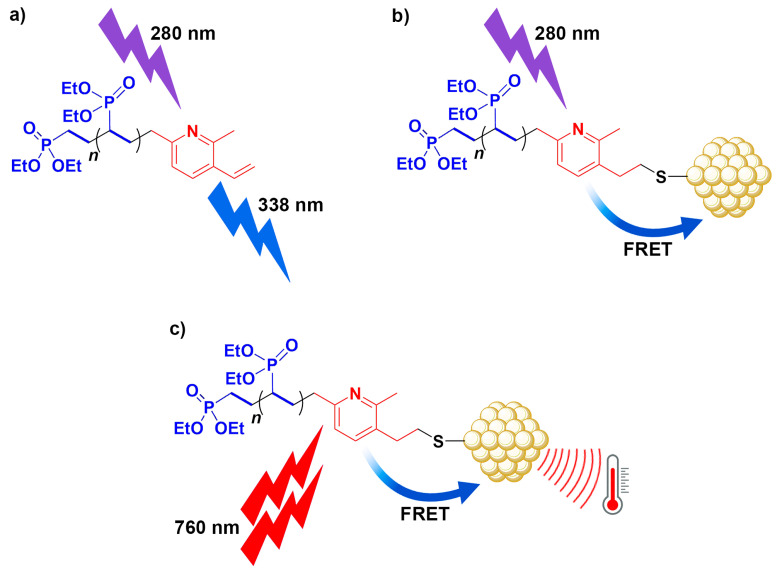
UV excitation and fluorescence emission of P(DEVP)-VL (**a**); UV excitation and fluorescence quenching of P(DEVP)-V-S-AuNP (**b**); NIR two-photon absorption and fluorescence quenching and thermal response of P(DEVP)-V-S-AuNP (**c**).

The WAXD spectrum of this sample does not show reflections for crystalline *fcc* gold. In Figure 4, the WAXD diffractogram of PVDEP-AuNPs is compared with the one of AuNPs embedded in PVDEP; this result suggests the presence of sub-nanometric gold nanoparticles in PVDEP-AuNPs. In agreement with this observation, the UV spectrum (Figure 5c) of the same sample does not show the intense band due to the plasmonic resonance of big (4–20 nm) AuNPs expected at about 520 nm [29]; two UV bands at 257 and 310 nm confirm the presence of lutidine units whose absorbance is red-shifted with respect to the same bands observed in 3-VL and PDEVPs, end-capped with lutidine at 240 and 280 nm and 240 and 284 nm, respectively (Figure 5a,b; for a comparison, the UV–Vis spectrum of PVDEP is shown in Figure S8). A red-shift of the UV–Vis absorption band of the chromophore characterizes the coordination of a chromophore to gold nanoparticles [36,37,38,39]. More interestingly, aqueous solution of PVDEP-AuNPs (0.4 mg/mL) excited at 280 nm exhibit the complete quenching of fluorescence expected at 338 nm because of the Förster resonance energy transfer (FRET) interaction between the metal nanoparticle surface and pyridine unit; actually, this emission is detectable in the spectra of both aqueous solution of 3VL and PDEVP end-capped with the same fluorophore (see Figure 5d–f and Figure 6). The complete fluorescence quenching of this chromophore can be attributed to the anchoring of the chromophore to gold nanoparticles. VL fluorescence would have been observed if polymer chains were not bound to the gold particles. The reason for this important result lies in the appropriate identification of the molar ratios between gold precursor and thioacetylated polymer chains. The addition of the gold precursor after sodium hydroxide-mediated deacetylation of PDEVP-VL-TA ensures the formation of gold-thiolates, and therefore, all polymer chains bearing the VL terminal are already anchored to gold. The subsequent addition of the reducing agent NaBH_4_ completes the reduction of gold to metallic gold.

### 3.4. PVDEP-VL-Capped AuNPs: Morphology and Thermoresponsive Behavior

**Figure 7:**
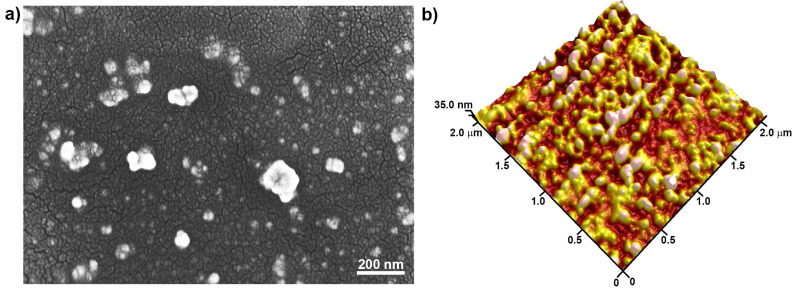
SEM (**a**) and TM-AFM (**b**) micrographs of P(DEVP)-VL-S-AuNPs.

The morphological analysis by scanning electron microscopy (SEM) and atomic force microscopy operating in tapping mode (TM-AFM) of the PVDEP-AuNPs, which were dispersed in water, deposed onto a glass slide, and dried in air at room temperature, evidenced the formation of spherical structures with sizes of 50–200 nm (see Figure 7).

**Figure 8:**
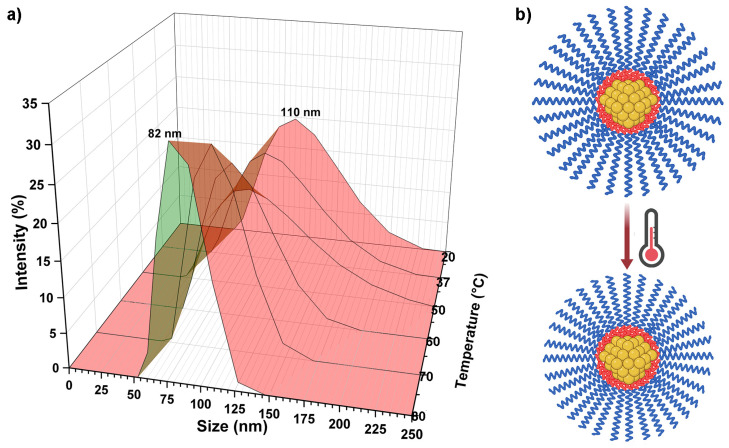
(**a**) Size distribution profiles of P(DEVP)-S-AuNPs in water (0.7 mg/mL) at variable temperatures determined by DLS (see additionally Figure S9). (**b**) Depiction of the thermoresponsive behavior of P(DEVP)-VL-S-AuNPs.

The hydrodynamic radius of the PVDEP-AuNPs in water (0.7 mg/mL) was determined by dynamic light scattering (DLS). The size distribution of the colloids in water media at 20 °C was broad and monomodal, found in the range 50–250 nm centered at 110 nm (see Figure 8 and Figure S9), in good agreement with the size found in the solid state by SEM and AFM analyses (Figure 7). The effect of the temperature on the hydrodynamic radius of the PVDEP-AuNPs was then investigated. Increasing the temperature, the average size decreased, and the size distribution of the colloids progressively became narrower: the average size at 80 °C was ≈80 nm, with overall size ranging from 50 nm to 130 nm. The thermoresponsive properties of PDEVP have been extensively studied by Rieger and collaborators [40]. The LCST for this polymer at high mass (>10^5^ Da) is between 45–50 °C, the same temperature where the contraction of PDEVP-VL-S-AuNPs particles begins (see Figure S9).

### 3.5. Photothermal Effect of PVDEP-VL-Capped AuNPs

PVDEP-VL-capped AuNPs exhibited photothermic properties when excited with an ultrafast pulsed laser. In particular, co-cultures of cortical neurons and astrocytes were incubated for 24 h with AuNPs conjugated with PDEVP (either 17 KDa and 25 KDa, Table 1) (at 700 µg/mL concentration in culture medium) to allow their internalization in neural cells. After incubation, cells were transferred in a microscope chamber filled with Tyrode’s solution and placed under a 40× objective of a confocal system connected to an ultrafast pulsed laser (see Section 2). Under illumination at 760 nm for 1.26 s, PDEVP-coated AuNPs developed sufficient heat to generate bubbles in the aqueous culture medium (see Buonerba et al., 2020 for comparison) [29] over a laser power estimated at 270 W/cm^2^ (or 340 J/cm^2^ fluence) on the sample. The VL chromophore acts as an antenna for the pulsed NIR radiation with a wavelength of 760 nm. The two-photon absorption of this pulsed light corresponds to absorption in the UV range; the emission from the chromophore is then quenched via FRET by the AuNP, and finally, the radiation is converted into heat useful for photoablation of cells (see Figure 6).

## 4. Conclusions

A new nanodevice with interesting photophysical and thermoresponsive behavior based on gold nanoparticles capped with poly(diethylvinylphosphonate) has been synthesized and characterized. The synthetic strategy involved synthesizing an Yttriocene catalytic system bearing a vinyl-lutidine functionality, where the latter acts as an initiator of the group transfer polymerization of diethyl vinyl phosphonate. The obtained polymer, bearing the terminal unsaturated functionality, was then thioacetylated, deacetylated, and finally reacted in situ with tetrachloroauric acid in the presence of sodium borohydride, leading to the one-pot synthesis of the PDEVP-VL-S-capped AuNPs. Spectroscopy characterization (NMR, UV–Vis, ESI-MS) confirmed the formation of the described synthetic intermediates, leading to the formation of the desired colloidal systems. WAXD and UV–Vis analysis highlighted the formation of subnanometer gold particles. The effective anchoring of the thiolated PDEVP to the AuNPs was confirmed via UV–Vis and fluorescence analysis. The terminal pyridinic fluorophore of PDEVP-VL-S is effectively anchored to the AuNPs, as its fluorescence emission (emission at 338 nm with excitation at 280 nm) was, in fact, quenched via FRET. Morphological analysis by SEM and AFM microscopy confirmed the formation of spherical structures with a size of 50–200 nm. This size was also confirmed in aqueous dispersion via DLS analysis. Finally, the study of the aqueous hydrodynamic radius of the PDEVP-VL-S-AuNPs colloidal systems evidenced the contraction of the colloids with the increase in temperature, confirming the thermoresponsive behavior of these new materials. These photophysical and thermoresponsive properties found in an aqueous dispersion of PDEVP-VL-S-AuNP colloids could have potential biomedical applications for the photoablation of malignant cells or the controlled delivery and release of drugs induced by light or thermally.

## Figures and Tables

**Table 1:** Synthesis of poly(diethyl vinyl phosphonate).

Entry	[DEVP]/[Y]	Yield	*M*_W_	DEVP
	(Molar Ratio)	(g)	(kDa)	(Units in Polymer Chain)
1	40	1.39	17.6	107
2	60	1.48	18.2	111
3	90	1.69	25.6	156

Reaction condition: C_1_/C_2_ (0.305 mmol, 0.129 g), toluene (20 mL), DEVP, 25 °C, 1 h.

## Data Availability

Data will be provided, on reasonable request, from the corresponding author.

## Supplementary Materials

The supporting information can be downloaded at https://www.mdpi.com/article/10.3390/nano14191589/s1. Figure S1. ^1^H NMR spectrum of 3-vinyl-lutidine (300 MHz, CDCl_3_, 25 °C). Figure S2. ^1^H-^1^H COSY NMR spectrum of P(DEVP)-VL (600 MHz, DMSO-*d*_6_, 90 °C). Figure S3. ^1^H-^13^C HSQC NMR spectrum of P(DEVP)-VL (600 MHz, DMSO-*d*_6_, 90 °C). Figure S4. ^13^C NMR spectrum of P(DEVP)-VL (600 MHz, DMSO-*d*_6_, 90 °C). Figure S5. ^13^C NMR spectrum of P(DEVP)-VL-TA (600 MHz, DMSO-*d*_6_, 90 °C). Figure S6. ESI-MS spectrum of the oligomer mixture obtained with Y/DEVP = 3. Figure S7. FT-IR spectrum of P(DEVP)-TA with labelled diagnostic band for thioacetyl functionality. Figure S8. FT-IR spectrum of P(DEVP). Figure S9. Size distribution profiles of P(DEVP)-S-AuNPs in water at variable temperatures determined by DLS (see additionally Figure 6).
