# Supplementary material for: Thermo- and Photoresponsive Smart Nanomaterial Based on Poly(diethyl vinyl phosphonate)-Capped Gold Nanoparticles
Source: Nanomaterials (Basel). 2024 Oct 1;14(19):1589. doi: 10.3390/nano14191589 (PMC11478069; doi:10.3390/nano14191589)
Supplement: Supplementary file 1 [file nanomaterials-14-01589-s001.zip › nanomaterials-3156872-supplementary.pdf]

Supporting Material

Thermo- and photo-responsive smart nanomaterial based on poly(diethyl vinyl phosphonate)-capped gold nanoparticles

Antonio Buonerba[1,2*], Rosita Lapenta[1], Francesco Della Monica[3], Roberto Piacentini[4], Lucia Baldino[5], Maria Rosa Scognamiglio[5], Vito Speranza[5], Stefano Milione[1,2], Carmine Capacchione[1,2], Bernhard Rieger[6], and Alfonso Grassi[1,2]

1 Department of Chemistry and Biology "Adolfo Zambelli", University of Salerno, via Giovanni Paolo II, 84084, Fisciano (SA), Italy.

2 CIRCC - Consorzio Interuniversitario per le Reattività Chimiche e la Catalisi, Villa La Rocca, Via Celso Ulpiani 27, 70126, Bari, Italy.

3 Department of Biotechnology and Life Sciences, University of Insubria, via Jean Henry Dunant 3, 21100 Varese, Italy.

4 Department of Neuroscience of Università Cattolica del Sacro Cuore, and Fondazione Policlinico Uni-versitario A. Gemelli IRCCS, Rome, Italy.

5 Department of Industrial Engineering, University of Salerno, via Giovanni Paolo II, 84084, Fisciano (SA), Italy.

6 WACKER-Lehrstuhl für Makromolekulare Chemie, Zentralinstitut für Katalyseforschung (CRC), Tech-nische Universitat München, Lichtenbergstraße 4, 85747 Garching, Germany.

* Correspondence: abuonerba@unisa.it.

1. NMR characterization

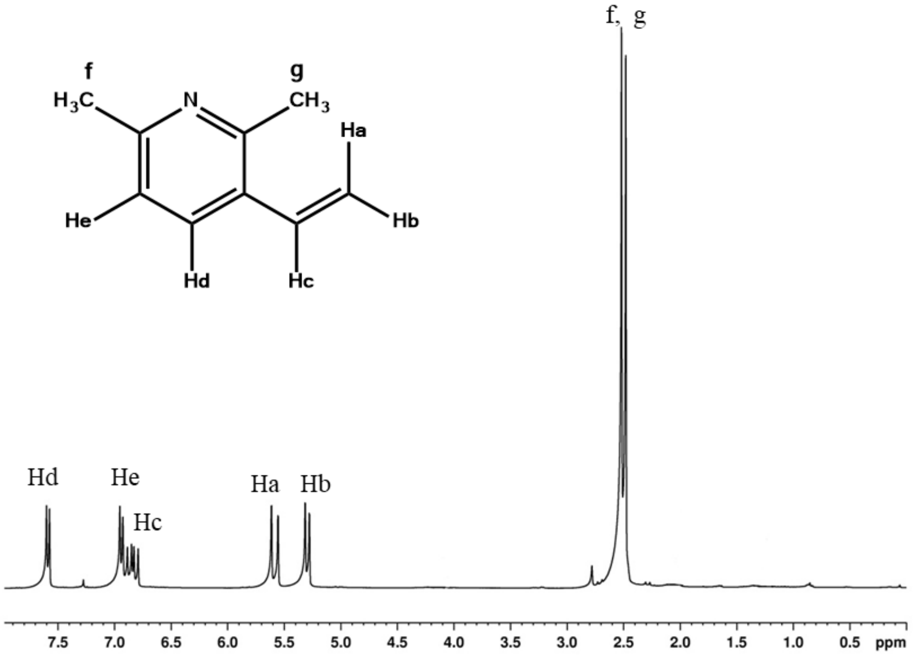

Figure S1. ^{1}H NMR spectrum of 3-vinyl-lutidine (300 MHz, $CDCl_3$, 25°C).

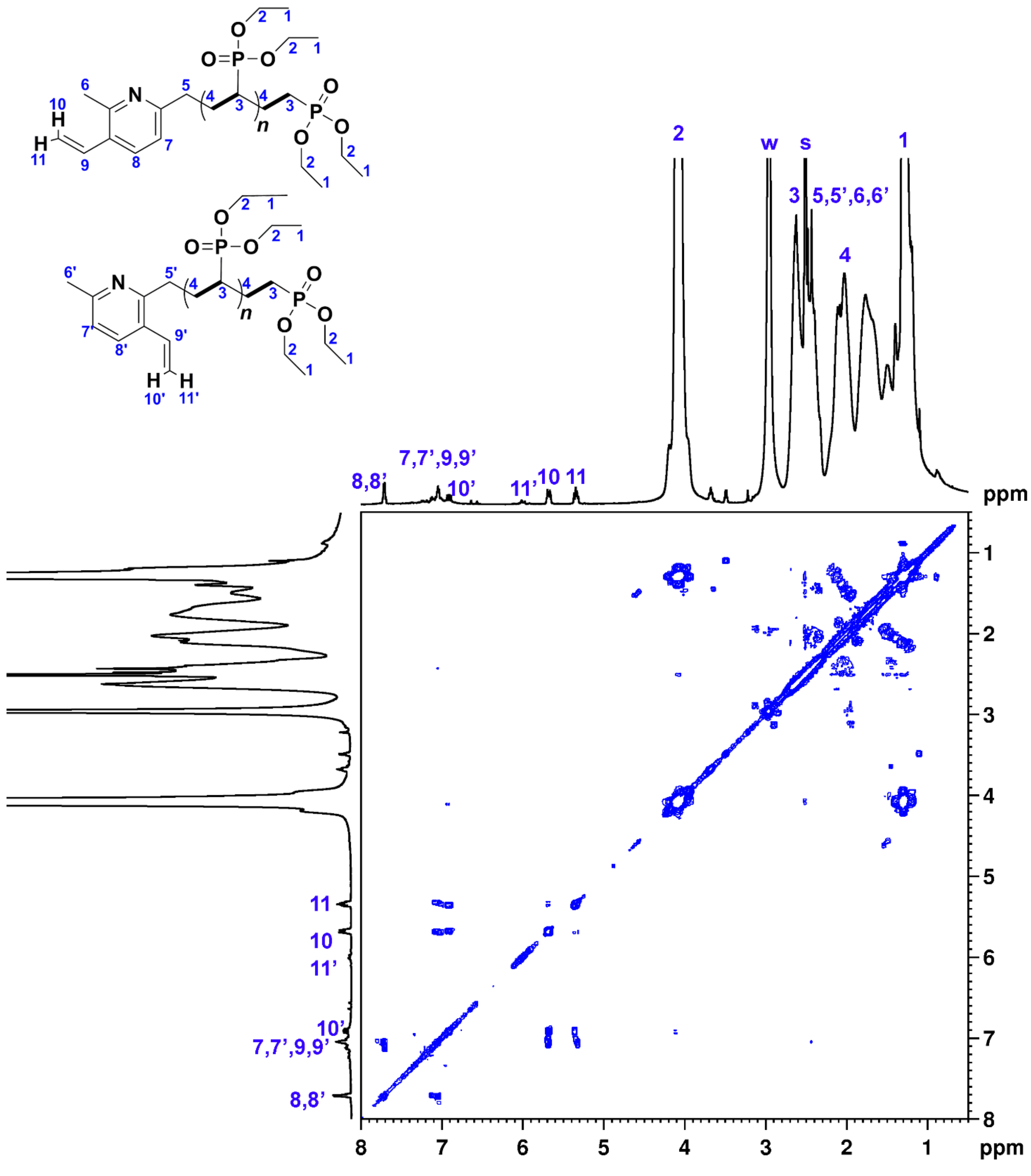

Figure S2. ^{1}H-^{1}H COSY NMR spectrum of P(DEVP)-VL (600 MHz, DMSO-d_6, 90°C).

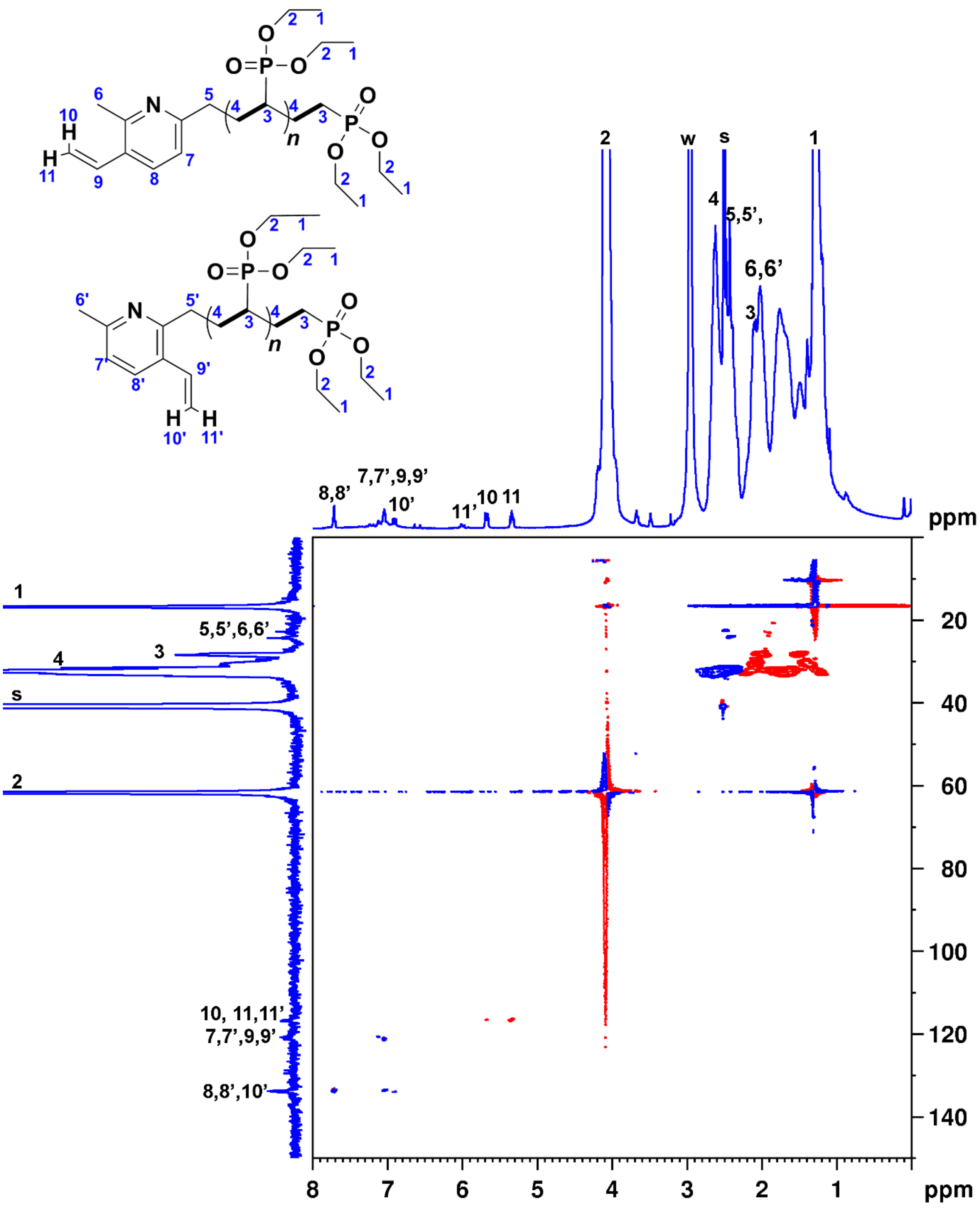

Figure S3. ^{1}H-^{13}C HSQC NMR spectrum of P(DEVP)-VL (600 MHz, DMSO-d_6, 90°C).

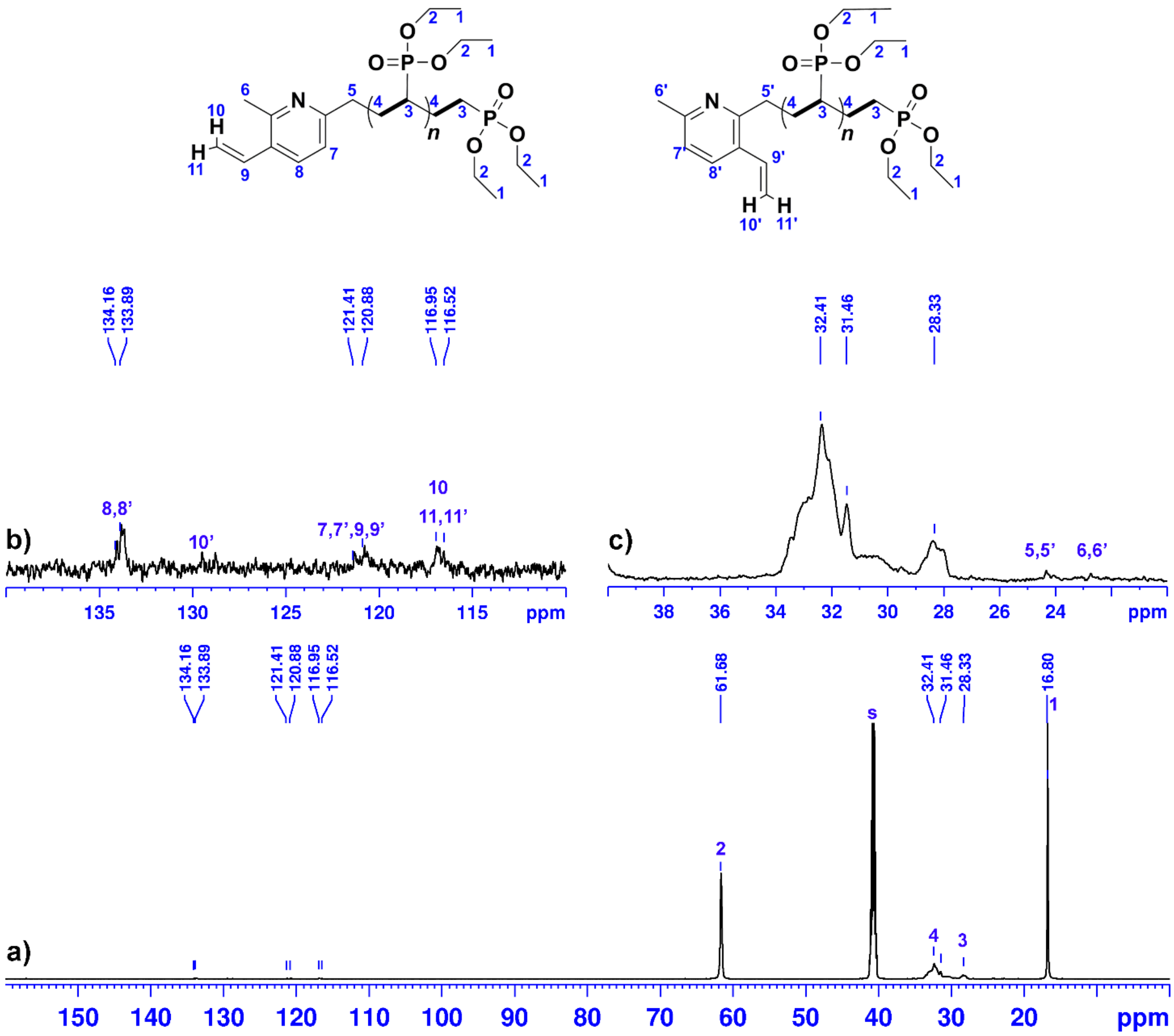

Figure S4. ^{13}C NMR spectrum of P(DEVP)-VL (600 MHz, DMSO-d_6, 90°C).

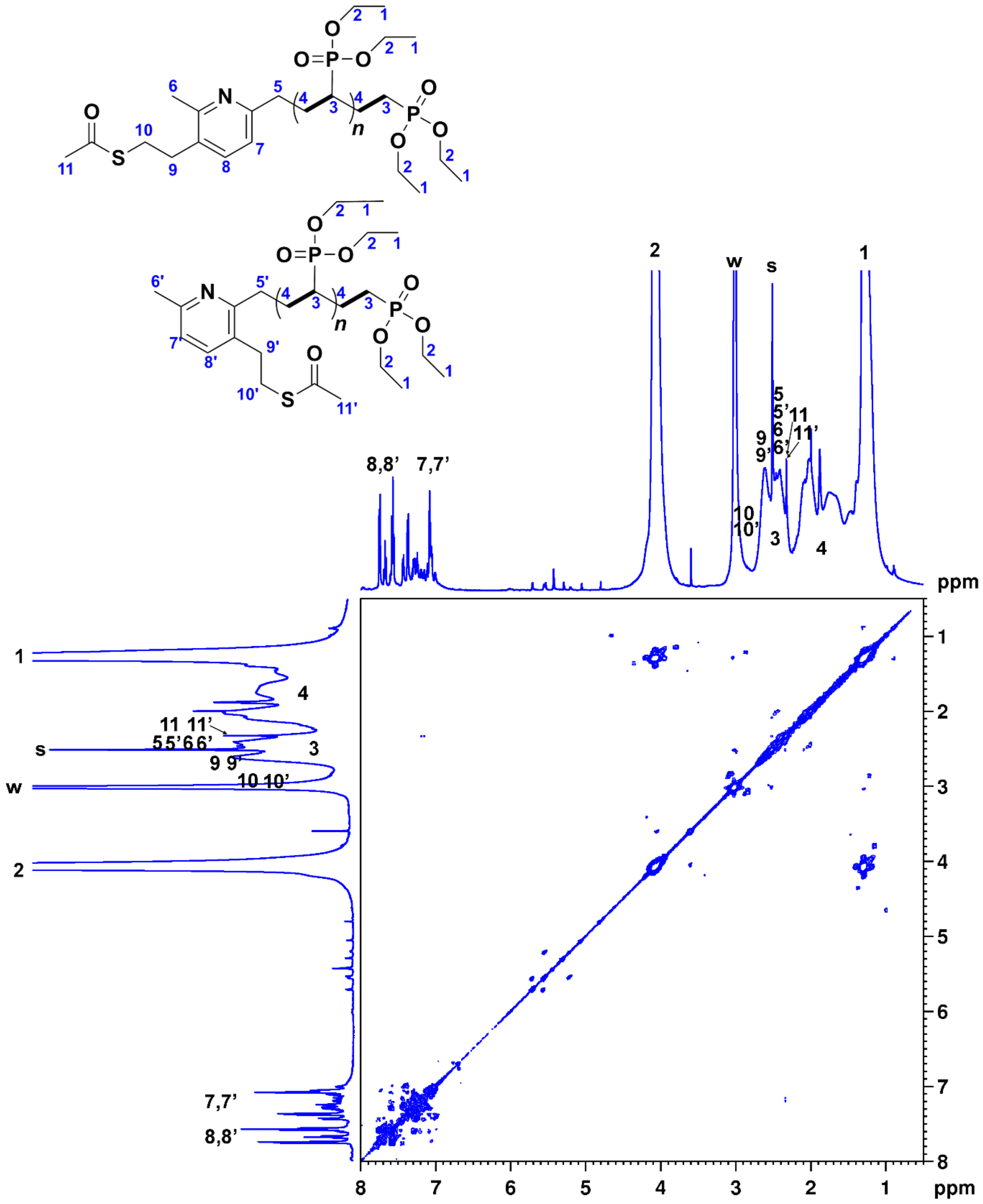

Figure S5. ^{13}C NMR spectrum of P(DEVP)-VL-TA (600 MHz, DMSO-d_6, 90°C).

2. ESI-MS characterization

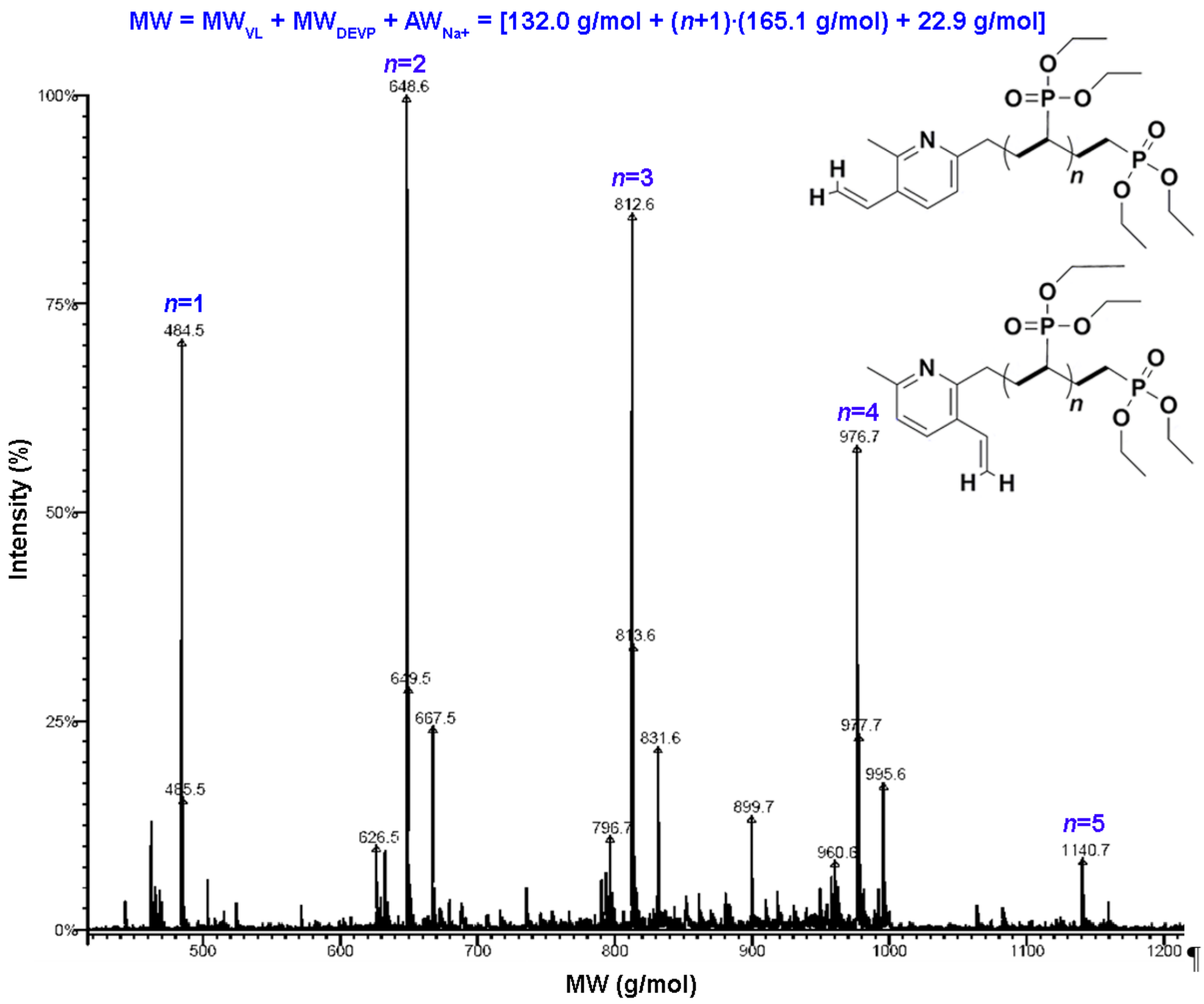

Figure S6. ESI-MS spectrum of the oligomer mixture obtained with Y/DEVP = 3.

3. FT-IR characterization

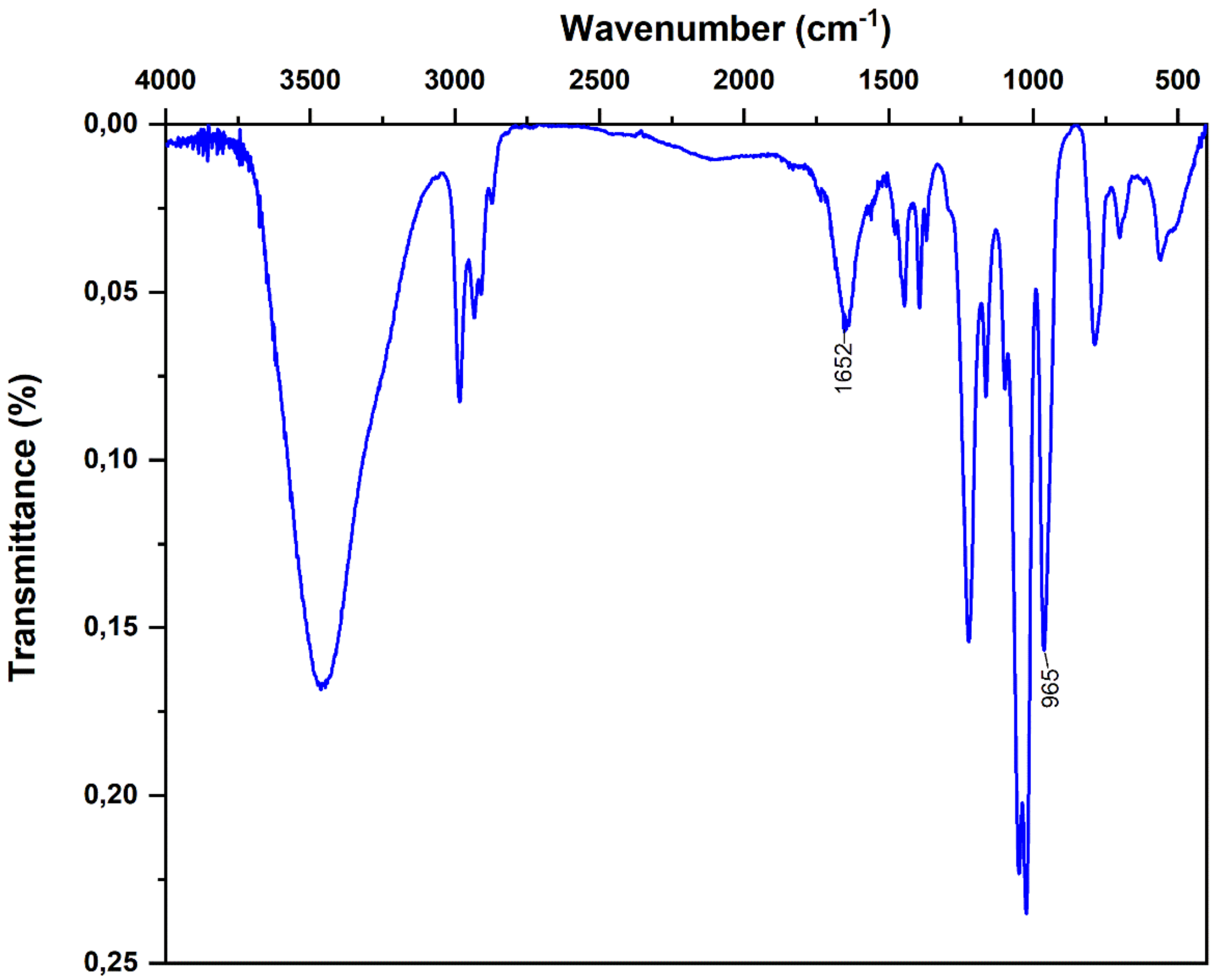

Figure S7. FT-IR spectrum of P(DEVP)-TA with labelled diagnostic band for thioacetyl functionality.

4. UV-Vis characterization

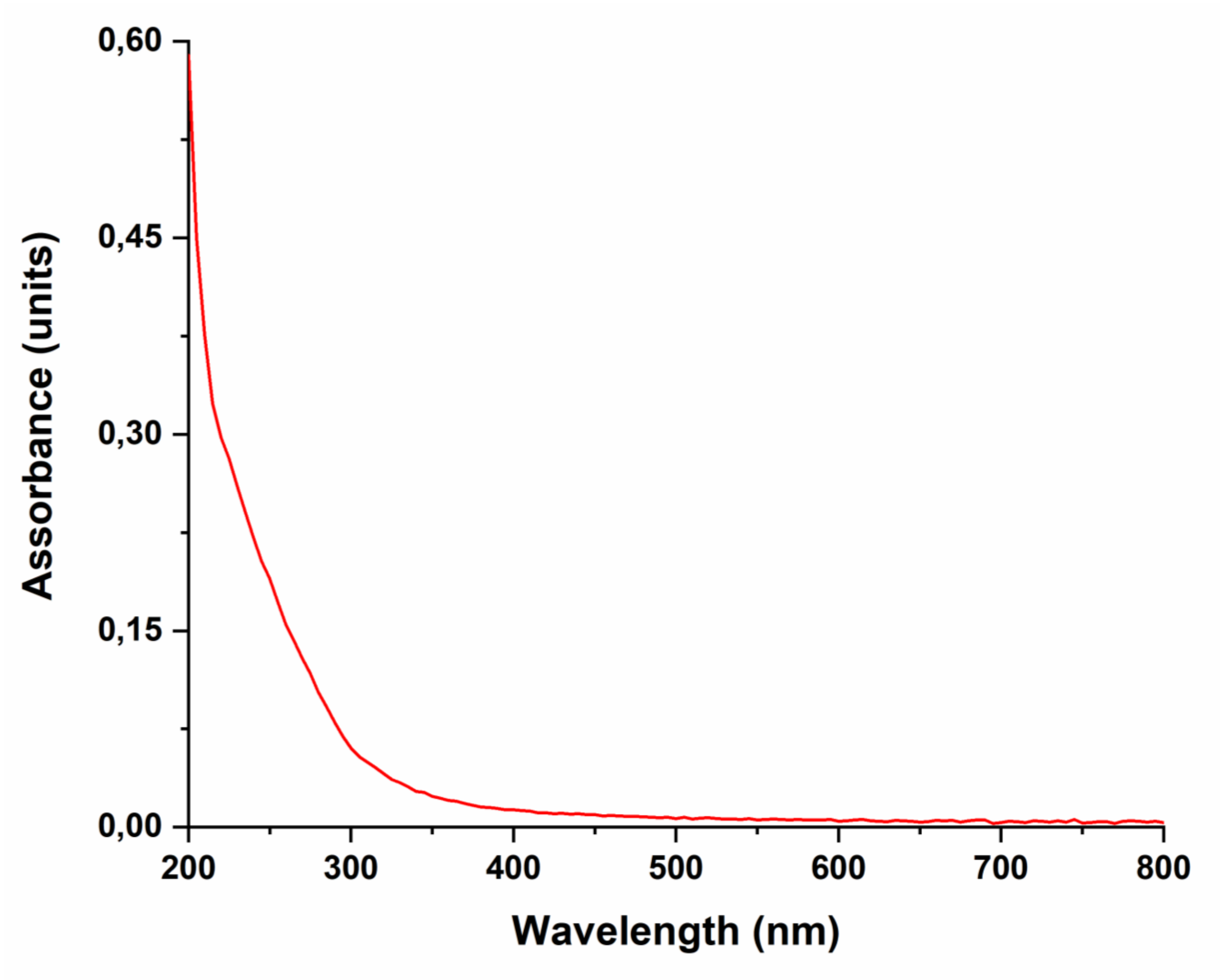

Figure S8. FT-IR spectrum of P(DEVP)**.**

1. DLS characterization

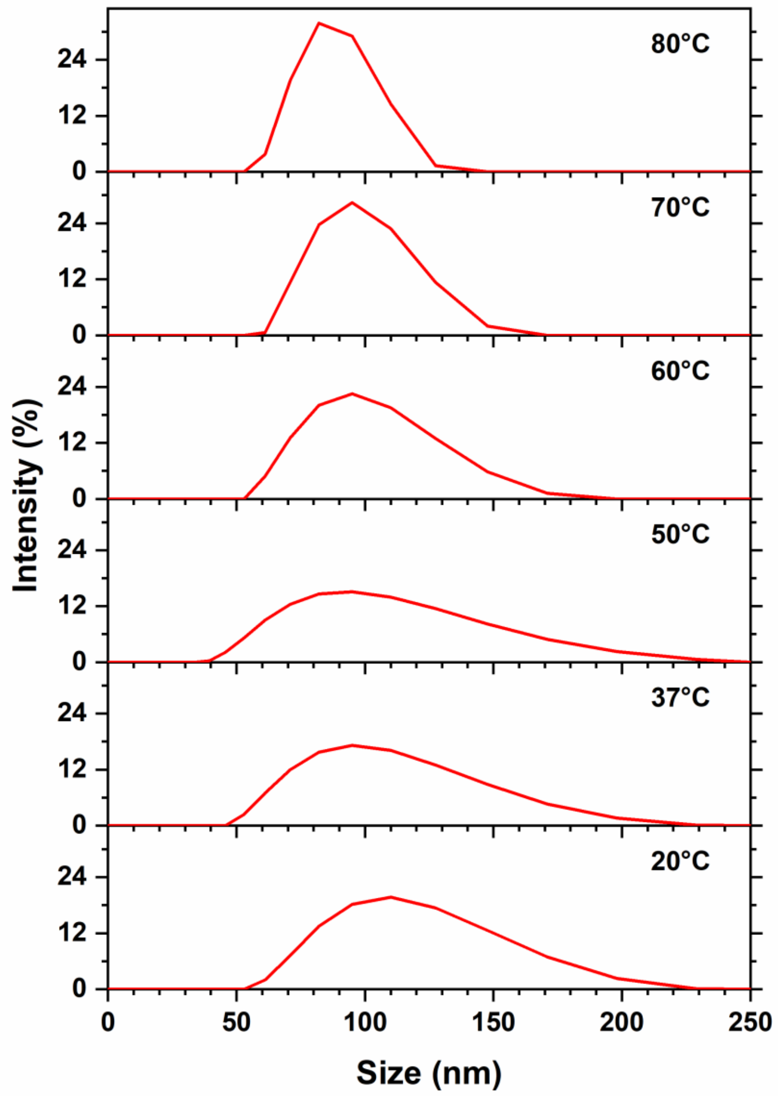

Figure 9. Size distribution profiles of P(DEVP)-S-AuNPs in water at variable temperatures determined by DLS (see additionally Figure 6).
